# Prevalence of amebiasis in inflammatory bowel disease in University Clinical Hospital Mostar

**DOI:** 10.1186/s40064-016-3261-7

**Published:** 2016-09-15

**Authors:** Emil Babić, Milenko Bevanda, Mladen Mimica, Maja Karin, Mile Volarić, Ante Bogut, Tatjana Barišić, Danijel Pravdić, Nikica Šutalo

**Affiliations:** 1 Department of Gastroenterology and Hepatology, University of Mostar Clinical Hospital, Bijeli Brijeg bb, 88000 Mostar, Bosnia and Herzegovina; 2 Department of Ginecology, University of Mostar Clinical Hospital, Bijeli Brijeg bb, 88000 Mostar, Bosnia and Herzegovina; 3 Department of Abdominal Surgery, University of Mostar Clinical Hospital, Bijeli Brijeg bb, 88000 Mostar, Bosnia and Herzegovina

**Keywords:** Amebiasis, Inflammatory bowel disease, Prevalence, Immune response, Inflammation

## Abstract

**Aim:**

To explore the prevalence of amebiasis in inflammatory bowel disease (IBD), Crohn’s disease and ulcerative colitis, in patients in Clinical hospital Mostar (Bosnia and Herzegovina, region of Herzegovina).

**Methods:**

In this study, *Entamoeba histolytica/dispar* prevalence was investigated in fresh faeces by native microscopy and immunochromatographic rapid assay “RIDA^®^QUICK *Entamoeba* test”, in 119 cases of new found IBD patients, 84 of ulcerative colitis and 35 of Crohn’s disease and in control group who had also 119 patients who didn’t have any gastrointestinal complaints. IBD diagnosis was established by standard diagnostic procedures (anamnesis, clinical manifestations, laboratory, endoscopy and biopsy).

**Results:**

*Entamoeba histolytica/dispar* were found in 19 (16.0 %) of a total of 119 cases, 12 (14.3 %) of the 84 patients with ulcerative colitis and 7 (20.0 %) of the 35 patients with Crohn’s disease. As for the 119 patients in the control group who had not any gastrointestinal complaints, 2 (1.7 %) patients were found to have *E. histolytica/dispar* in their faeces. Amoeba prevalence in the patient group was determined to be significantly higher in group with Crohn’s disease, ulcerative colitis and IBD total than in the control group (p < 0.001).

**Conclusion:**

Ameba infections in patients with Crohn’s disease and ulcerative colitis, have a greater prevalence compared to the normal population.

## Background

Amebiasis affects around 500 million people in the world today (Andersen 2000). It is more prevalent in developing countries (Lau et al. 2013; Verma et al. 2012). The prevalence of parasite infections and amebiasis is very high in Mediterranean region (Abdulsalam et al. 2013; Ozçelik et al. 2012; El Guamri et al. 2011). Symptoms of amebiasis can overlap with symptoms of the inflammatory bowel disease (IBD). It can leads to difficulty in diagnosis and treatment in IBD (Hansen and Lund 1998). In that case the diagnosis and management of inflammatory bowel disease can be challenging as certain infections can mimic IBD and lead to a misdiagnosis. Because of the increasing use of corticosteroids, immunosuppressive drugs and biological agents the risk of opportunistic infection including amebiasis are also higher in IBD patients. The role of the physician lies not only in the diagnosis and management of IBD but also in the ability to prevent, recognize and treat infections.

*Entamoeba histolytica and Entamoeba dispar* are intraluminal parasites. *Entamoeba histolytica* is invasive species and cause symptomatic disease characterized with abdominal pain, cramps, blood diarrhea. *Entamoeba dispar* is non-invasive species which is useful as a study model for *E. histolytica* because both species have a lot of identical gens regions and similar immunogenic effect (Willhoeft et al. 1999; Bruchhaus et al. 1996; Jacobs et al. 1998). IBD is a chronic inflammatory condition of the intestines caused by inadequate mucosal immune response to antigenic components. It is manifested in two major subtypes: Crohn disease and ulcerative colitis. A consensus hypothesis is that in genetically predisposed individuals, a combination of factors including luminal flora and host characteristics result in a chronic state of dysregulated mucosal immune function. This results in an “inappropriate response” to normal microbial flora within the intestine, with or without some component of autoimmunity (Friedman and Blumberg 2008).

Amebiasis can exacerbate symptoms of IBD and has unfavorable influence on course of disease and therapy. Inadequate mucosal immune response on the intraluminal antigenic components is essential in IBD pathogenesis (Kaser et al. 2010).

In this study, the prevalence of *Entamoeba histolytica/dispar* in the patients hospitalized in Clinical hospital Mostar in Herzegovina region with predominantly Mediterranean climate, diagnosed with either Crohn’s disease or ulcerative colitis was investigated.

## Methods

IBD were diagnosed in 119 patients by clinical presentation, laboratory and serological, endoscopic and histopathologic examinations at Department of internal medicine, University clinical hospital Mostar between March 2009 and December 2011. All patients were treated in gastroenterology ambulance and clinic and IBD (Crohn disease and ulcerative colitis) were confirmed by standard procedure. There was 84 patients diagnosed as ulcerative colitis (70.6 %) and 35 as Crohn’s disease (29.4 %).

Also, 119 patients in the control group was formed by healthy individuals, without gastrointestinal complains and have regular medical examinations which include laboratory examinations and native microscopic exam of fresh feces samples. Therefore, control group was not matched in any way with IBD patients, except of course by age and gender (Tables 1, 2).

**Table 1:** Age distribution of male patients and controls

Age (years) (X ± SD)	Male patients	Control	t*	p
<37	28.27 ± 5.05	29.87 ± 5.82	0.885	0.382
38–52	45.10 ± 4.16	47.00 ± 4.31	1.381	0.176
≥53	63.95 ± 8.22	62.11 ± 8.42	0.682	0.5

* Student *t* test

**Table 2:** Age distribution of female patients and controls

Age (years) (X ± SD)	Female patients	Control	t*	p
<37	27.62 ± 5.45	29.96 ± 5.23	1.483	0.145
38–52	46.44 ± 4.55	46.42 ± 3.74	0.022	0.983
≥53	66.32 ± 7.81	62.94 ± 7.39	1.347	0.187

* Student *t* test

If native microscopy was found *Entamoeba hystolitica/dispar* cysts or trophozoits, immunochromatographic rapid assay “RIDA^®^QUICK Entamoeba test” is used to confirm diagnosis. Native microscopy is performed by the optical microscope, enlargement factor 10× and 40×. Fresh faeces samples taken from people were examined immediately using the wet mount, Lugol’s iodine and physiological solution. χ^2^ test was applied to the groups (ulcerative colitis, Crohn’s disease and control) for a comparison of amoeba frequency among them.

## Results

*Entamoeba histolytica/dispar* cysts and trophozoits were found in 19 (16 %) of the 119 IBD cases and in 2 cases of the control group (1.7 %) (Table 3). Frequency of *E. histolytica/dispar* in patients with IBD was significantly higher than in control group (Chi square test, p < 0.001). When the groups of patients with IBD were compared with the control group separately, the frequency of *E histolytica/dispar* in patients with ulcerative colitis and Crohn’s disease were also significantly higher than in the control group. *E. histolytica/dispar* were determined in 12 (14.3 %) of the 84 patients with ulcerative colitis and in 7 (20 %) patients with Crohn’s disease (Table 4).

**Table 3:** Prevalence of amebiasis in IBD and control group

*Entamoeba histolytica/dispar*	N (%)	
	IBD	Control group
Negative	100 (84.0)	117 (98.3)
Positive	19 (16.0)	2 (1.7)

χ^2^ = 13.37, p < 0.001

χ^2^ = Chi square test

**Table 4:** Prevalence of amebiasis in ulcerative colitis and Crohn’s disease

*Entamoeba histolytica/dispar*	N (%)		
	UC	CD	Control group
Negative	72 (85.7)	28 (80.0)	117 (98.3)
Positive	12 (3.14)	7 (20.0)	2 (1.7)

χ^2^ = 16.194, p < 0.001

*UC* ulcerative colitis, *CD* Crohn’s disease, χ^2^ = Chi square test

Demographics results about comparison between IBD, amebiasis and control group are shown in Table 5.

**Table 5:** Prevalence of amebiasis by age group of patients with IBD and the control group

Age (years)	Broj (%)				
	Amebiasis	IBD	Control	x^2^	p
≤37				9.676	0.002
	Negative	32 (74.4)	40 (100.0)		
	Positive	11 (25.6)	0 (0.0)		
Total		43 (100)	40 (100)		
38–52				4.492	0.049*
	Negative	32 (84.2)	41 (97.6)		
	Positive	6 (8.15)	1 (2.4)		
Total		38 (100)	42 (100)		
≥53				0.320	1.000*
	Negative	36 (94.75)	36 (97.3)		
	Positive	2 (3.5)	1 (2.7)		
Total		38 (100)	37 (100)		

* Fisher test

There was not significant difference between male and female patients with IBD and amebiasis compared with the control group and it was not found that gender is risk factor for amebiasis in IBD (Chi square test, p = 0.014 and p = 0.021). *E. histolytica/dispar* cysts and trophozoits were found in 11 (18.0 %) of the 61 IBD cases in male and 8 (13.8 %) of the 58 IBD cases in female.

Comparing results with previous studies, our results show higher prevalence of amebiasis in CD (Table 6).

**Table 6:** Study prevalence comparison

Study	Number (%)		
	UC	CD	IBD
Babic et al.	12/84 (3.14)	7/35 (20)	19/100 (16)
Ustun et al.	13/130 (10)	1/30 (3.3)	14/160 (8.75)
Tozum et al.	284/854 (33)	24/234 (10)	308/1088 (28.3)

χ^2^ = 11.55, p = 0.0031

*UC* ulcerative colitis, *CD* Crohn’s disease, χ^2^ = Chi square test

## Discussion

*Entamoeba histolytica/dispar* were determined in 19 (16 %) of the 119 patients with IBD and it was significantly higher than in control group (1.7 %). *E. histolytica/dispar* were determined in 12 (14.3 %) of the 84 patients with ulcerative colitis. Results from our study showed higher incidence than study of Prokopowicz et al. performed in Poland where prevalence of amebiasis in ulcerative colitis was five out of 103 patients which accounts for 4.85 % (Prokopowicz et al. 1994). Studies performed in Turkey were found higher prevalence of amebiasis up to 69 %. Bayramicli et al. were found the presence of amebiasis in 19 patients with diagnosis of ulcerative colitis and found amebiasis in 69 % oft he cases (Bayramicli et al. 1997). In a study they carried out by Suleymanlar et al. found *E. histolytica* cysts and trophozoites in 22 (54 %) oft he patients (Suleymanlar et al. 1996). Prevalence of amebiasis in ulcerative colitis seemed higher in countries with Mediterranean than continental climate. It is important to distinguish amebic colitis from IBD especially if amebiasis is present in the community or when the patient has visited an endemic area. Similarity in the symptomatology of these two diseases and the non-specific endoscopic findings can cause problem in diagnosis. Absence of amebic trophozoites and cysts in the stool in some cases of amoebic colitis can complicate diagnostic procedure because both amebic colitis and IBD may present with similar symptoms like bloody diarrhoea, abdominal pain, haematochezia, anaemia and hypoproteinaemia. Continuous mucosal inflammation typical of ulcerative colitis can be seen also in amebic colitis. We were found *E. histolytica/dispar* in 20.0 % with Crohn’s disease patients and it is higher than results Ustun and Tozum in Turkey where *E. histolytica/dispar* were found in only 13.3 and 10 % Crohn’s disease patients (Ustun et al. 2003; Tözün 2002). The reason for higher incidence of amebiasis in our study could be the fact that amebiasis is not recognized as public health problem in our country yet and the incidence of *E. histolytica/dispar* has been diminishing in Turkey in recent years. Not only amebic colitis and IBD may present with similar symptoms but endoscopic findings may be non-specific. For example, typical discrete flask-shaped ulcers of amebic colitis may also be seen in Crohn’s disease. It may be impossible to distinguish between the amebiasis and Crohn’s disease, since stool specimens, bowel biopsies, and serological studies may be negative for *Entamoeba histolytica*, even in the presence of invasive amoebic colitis. Diagnosis in such cases can be established only by pathological examination of a surgically resected bowel. That’s why is important to consider anti-amebic therapy in endemic areas in cases of persistent IBD (Lysy et al. 1991). Adding to the difficulty in differentiating amebic colitis from IBD is the possibility of not seeing the amebic trophozoites and/or cyst in the stool in acute invasive colitis with diarrhoea, and the fact that serologic test are not readily available in all hospitals, especially in our country. The pathological diagnosis of inflammatory bowel disease is often difficult because biopsy material may not contain pathognomonic features, making distinction between Crohn’s disease, ulcerative colitis and other forms of colitides a truly challenging exercise. The best way for reliant diagnosis is multidisciplinary approach when the full clinical history, endoscopic findings, radiology and relevant serology and microbiology are available (Woodman et al. 2015; Şimşek et al. 2016). This can be very important in patients with IBD because early anti-amebic therapy is essential and significantly influence on the course of disease. For example, in the study by Vinayak et al. trophozoites of *Entamoeba histolytica* were seen in less than 40 % of stool microscopy in patients with invasive intestinal disease (Vinayak et al. 1993). It is difficult to differentiate between infection. In our study, we found CRP significantly high in patients with comorbidity of IBD and amebiasis. Other studies confirmed CRP as good parameter of inflammation and disease activity in IBD or amebiasis separately but we didn’t find data about correlation in CRP and both disease simultaneously (Kiss et al. 2011; Ahmed et al. 1992). This result indicates on necessity of future investigations about this. We did not found that amebiasis is gender correlated and prevalence was not significantly higher in males or females in IBD.

## Conclusion

Results in our study indicate that differential diagnosis is extremely important for IBD and amebiasis patients, especially in region with high prevalence of amebiasis. It is essential to distinguish amebiasis colitis and IBD already on beginning of diagnostic algorithm (Ibrahim et al. 2005). Similarity in the symptoms of these two diseases can complicate diagnostic procedure because both may present with similar symptoms. It is very important to considered amebic colitis in cases of exacerbation of symptoms in inflammatory bowel disease in region with high prevalence of amebiasis.

Our results confirmed possibility that decrease of protective factors in IBD (slim in bowels, loss of albumins, inappropriate nutrition) enables colonization by parasites, including *E. histolytica/dispar* (Vucelic 2002). There is hyperactivity of mucosal immune system to the intraluminal antigens in IBD where *E. histolytica/dispar* can participate in initiation or maintenance of immune response.
